# Engineering Materials and Devices for the Prevention, Diagnosis, and Treatment of COVID-19 and Infectious Diseases

**DOI:** 10.3390/nano13172455

**Published:** 2023-08-30

**Authors:** Jennifer Soto, Chase Linsley, Yang Song, Binru Chen, Jun Fang, Josephine Neyyan, Raul Davila, Brandon Lee, Benjamin Wu, Song Li

**Affiliations:** 1 Department of Bioengineering, University of California Los Angeles, Los Angeles, CA 90095, USA; 2 School of Biomedical Engineering and Med-X Research Institute, Shanghai Jiao Tong University, Shanghai 200240, China; 3 Department of Dentistry, University of California Los Angeles, Los Angeles, CA 90095, USA; 4 Department of Medicine, University of California Los Angeles, Los Angeles, CA 90095, USA; 5 Eli and Edythe Broad Center of Regenerative Medicine and Stem Cell Research, University of California Los Angeles, Los Angeles, CA 90095, USA; 6 Jonsson Comprehensive Cancer Center, David Geffen School of Medicine, University of California Los Angeles, Los Angeles, CA 90095, USA

**Keywords:** biomaterials, biofabrication, point-of-care diagnostics, disease modeling, organ-on-a-chip, 3D printing

## Abstract

Following the global spread of COVID-19, scientists and engineers have adapted technologies and developed new tools to aid in the fight against COVID-19. This review discusses various approaches to engineering biomaterials, devices, and therapeutics, especially at micro and nano levels, for the prevention, diagnosis, and treatment of infectious diseases, such as COVID-19, serving as a resource for scientists to identify specific tools that can be applicable for infectious-disease-related research, technology development, and treatment. From the design and production of equipment critical to first responders and patients using three-dimensional (3D) printing technology to point-of-care devices for rapid diagnosis, these technologies and tools have been essential to address current global needs for the prevention and detection of diseases. Moreover, advancements in organ-on-a-chip platforms provide a valuable platform to not only study infections and disease development in humans but also allow for the screening of more effective therapeutics. In addition, vaccines, the repurposing of approved drugs, biomaterials, drug delivery, and cell therapy are promising approaches for the prevention and treatment of infectious diseases. Following a comprehensive review of all these topics, we discuss unsolved problems and future directions.

## 1. Introduction

COVID-19 is a respiratory disease caused by the severe acute respiratory syndrome coronavirus 2 (SARS-CoV-2) that was discovered in the fall of 2019 and has since resulted in a global pandemic [1,2,3], affecting individuals across 220 countries around the world with more than 700 million confirmed cases and over 6 million deaths [4]. Though transmission is mainly thought to occur via the spread and contact of respiratory droplets from an infected individual [5,6], other methods of transmission, including being airborne, are still under investigation [7,8,9]. SARS-CoV-2 has been found to be closely related to SARS-CoV, sharing an 89.8% sequence identity in their spike (S) protein [10], a viral component that facilitates viral binding and entry into target cells. Similar to SARS-CoV, both viruses utilize the human angiotensin-converting enzyme 2 (ACE2) receptor as a mechanism to bind and infect human cells [11,12,13,14]. SARS-CoV-2 spike protein, however, has a higher binding affinity to ACE2 receptors [15], which accounts for its higher infectivity and transmissibility. Upon engaging with the receptor, plasma-membrane-associated serine proteases such as transmembrane protease serine 2 (TMPRSS2), TMPRSS4, and furin prime the S protein, enabling the fusion of viral and cellular membranes and promoting virus entry into host cells [13]. After an incubation period of approximately 4–5 days following infection [16,17], there may be development of symptoms in individuals, although an unknown proportion of infected individuals appear to be asymptomatic [18,19], potentially contributing to the widespread transmission of the virus unknowingly. The most common symptoms associated with COVID-19 infection include fever, dry cough, fatigue, shortness of breath, loss of smell or taste, and headaches [20]. As symptoms progress in severity, some patients may develop severe pneumonitis, “cytokine storm” (i.e., hyperinflammation), intravascular coagulation, and thrombotic complications [21,22,23,24]. In cases where the disease severity reaches a critical stage and patients are not able to recover through medical intervention, further complications such as acute respiratory distress syndrome (ARDS), systemic inflammatory response syndrome (SIRS)/septic shock, and multi-organ failure can arise that are ultimately fatal. While SARS-CoV-2 predominantly targets the lung, other organs such as the gastrointestinal tract, kidney, and cardiovascular system can also be affected [21]. Besides the need for specific antiviral drugs against COVID-19 infection, it is essential to develop technologies for effective prevention, accurate and timely diagnosis, and novel therapies to treat patients.

Recent advancements in the fields of micro and nano technologies, materials sciences, and bioengineering, including but not limited to three-dimensional (3D) printing, new imaging technologies, portable and rapid disease diagnostics, in vitro cell culture systems for disease modeling, automated and high-throughput systems for biopharmaceuticals, and novel biomaterials, can serve as powerful tools to combat COVID-19 and many other infectious diseases. In this review, we will present and discuss micro/nano technologies, materials, and bioengineering approaches that can be utilized for the fabrication of protection equipment, detection and diagnosis, disease modeling, drug discovery, and therapeutic development by using COVID-19 as an example, although these findings can be generalized for broad applications in the future to fight infectious diseases. From the design and production of equipment critical to first responders and patients using three-dimensional (3D) printing technology to computed tomography imaging and point-of-care devices for rapid diagnosis, these technologies and tools have been essential to address current global needs. Moreover, organ-on-a-chip platforms provide a valuable platform for disease modeling and drug screening, whereas biomaterials, drug delivery, and cell therapy are promising approaches for the prevention and treatment of infectious diseases.

## 2. Three-Dimensional Printing of Protection Equipment

**Figure 1:**
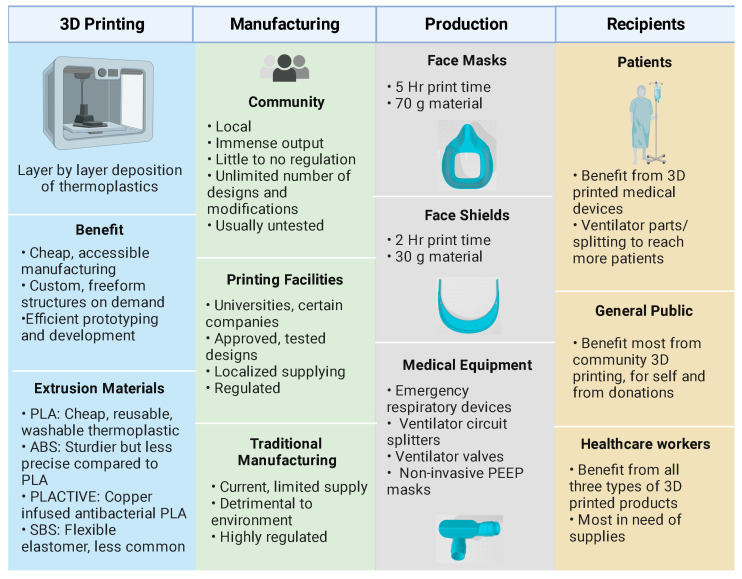
Applications and benefits of 3D printing during the COVID-19 pandemic. Three-dimensional printing can be utilized to address critical supply shortages, such as personal protective equipment and medical devices, which benefit patients, healthcare workers, and the community.

The COVID-19 pandemic led to sudden worldwide shortages of medical equipment and supplies, including personal protective equipment (PPE), that put healthcare workers and patients at extreme risk [25]. As a result, institutions resorted to decontaminating and reusing PPE, relying on community donations, and stretching individual units across multiple patients. In response, communities of academics, businesses, hobbyists, and manufacturing experts came together to develop open-source computer-aided design (CAD) models of 3D-printed equipment and supplies (Figure 1). The open-source platforms used by these creators allowed for collaborative work and produced increasingly fine-tuned products in extremely short timelines [26]. Although stereolithography (SLA) 3D printing is frequently used in the medical field for fabricating products using biocompatible materials, the ubiquity of fused deposition modeling (FDM) 3D printers allowed for local manufacturing on desktop 3D printers, which increased the impact of the technology [26]. This technology produces custom, freeform parts through the layer-by-layer deposition of thermoplastic material [26]. Though 3D printing can be utilized to generate many essential products, such as air exchangers, filter adaptors, in-line filter housing, and pneumotachometers, 60% of COVID-19 print projects in 2020 were solely for PPE such as reusable face masks and face shields [27]. 

### 2.1. Materials for 3D-Printed PPE

To classify as functional PPE, printed masks must fit on a wide range of face shapes yet be tight enough to form an adequate seal. Two studies found significant differences in the effectiveness of one-size-fits-all face masks and suggested providing at least three distinct sizes. Looking at face shape data of a large population, they concluded the most important facial dimensions to consider in design are bigonial breadth, bizygomatic breadth, menton–sellion length, and nose protrusion [28,29]. Another group confirmed insufficient seals are almost always around the nose when wearing printed masks. To combat this, customized face mask production is possible by 3D scanning the wearer’s facial parameters. Free applications, such as Bellus3D FaceApp, are available to download on devices with two cameras [30]. The seal performance of custom face masks significantly improves protection, but this individualized approach limits manufacturing throughput [30,31]. Others avoid customization by printing with flexible, elastomeric materials such as styrene–(ethylene–butylene)–styrene (SEBS) or a polypropylene (PP) blend. In these cases, mask design mimics commercial N95 respirators where one solid piece of nonwoven filtration material is designed to seal to the user’s face [32,33]. SEBS can be extruded at low temperatures with low risk of distortion, and PP is a low-cost, high-integrity material [32,34]. Rather than shape and fit, engineering lies in controlling blend ratios and nozzle temperature, as these parameters will influence the flexibility and the resulting seal. Another flexible option is electrospun nanofiber web (ESW) with polyacrylic blends. With a small pore size of around 270 nm and a large specific surface area, it can easily obtain removal efficiencies of 99.994% against tests with 300–500 nm NaCl aerosol particles, easily comparable to N99 masks [34]. However, it is too fragile to be printed into a mask or small filter alone. One group successfully deposited ESW onto a supporting mesh or fabric substrate to improve its mechanical properties while retaining its filtering properties [34]. Furthermore, because ESW is transparent, it is particularly useful in lip reading situations for the impaired. The material was the clearest when printed at 210 °C [34]. Unfortunately, such sophisticated materials are not available to the general public with a small desktop FDM 3D printer.

Instead, the most common thermoplastic filaments, polylactic acid (PLA) and acrylonitrile butadiene styrene (ABS), are hard plastics when cooled. With hard plastics, the mask usually requires up to two printed reusable components—one creating the seal against the face and one to secure the filter, as well as two disposable components—head straps to fix the mask to the wearer and a small filter piece [30]. These masks are cheap to produce (e.g., USD 1–2 per mask), and their durability enables them to survive multiple reuses, unlike SEBS/PP or ESW [35]. Additionally, most take advantage of the materials’ thermal properties to “customize” the fit by using hot water or a hair dryer to soften the hard plastic so it can be molded to fit the wearer’s face. The design of most hard plastic masks directs airflow through a small filtration opening to reduce the size of filtration material required. It is usually a piece of PP microfiber that comprises N95 masks. However, because of its large pore size and fiber diameter, the piece had to be at least 1 cm thick for sufficient filtration [35]. This thickness combined with the small ventilation hole in these 3D-printed masks can result in breathing discomfort and air leaks through the sides, comprising its functionality. Instead, MERV16 filters have been suggested for their high filtration and low cost. One group deduced that two layers of MERV16 sandwiched between two layers of MERV13 material resulted in the highest filtration [35]. The MERV13 layers were used as a protective measure, as MERV16 filters might contain fiberglass [35]. Around 1000 sets of filter cartridges can be made from each sheet, bringing the per-mask cost to around USD 0.10 [35]. One group improved upon the ESW method by printing it with PLA after fine-tuning nozzle temperatures [34]. The sturdier material improved ease of handling as well as dramatically reduced the cost. In this case, the nozzle temperature was a crucial parameter to influence material morphology from typical PLA properties [34]. The higher temperatures allowed for higher tensile strength due to better fusion between layers, while not affecting fiber structure. The researchers found that at 230 °C, filtration efficiency increased from 66.32 wt.% to 95.24 wt.% [34]. 

Along with trying to replicate the filtering properties of N95 masks, optimizing throughput is also a key component to designing PPE meant to be 3D printed. Even with dedicated 3D printing labs that can run many large printers at once and that can support up to three masks per bed, the time required for each unit is still the biggest bottleneck to production, as a single mask may require several hours [30,35]. One group tested many popular face shield and mask designs for the amount of material and time needed to print and found that, on average, face shield production is twice as fast and needs half as much material than face masks [27]. Face shields simply require a printed strap that wraps around the head with fasteners to which a transparent sheet of plastic can be attached. The clear plastic can also be printed through synthetic polymers such as polycarbonate/polyester and polyvinyl chloride if available [36]. Due to its resulting low production cost, labs prefer printing face shield supports over masks. To meet the demands for face masks, innovative designs that optimize throughput while preserving function still require further investigation. 

### 2.2. Safety and Quality Considerations

Regardless of the part being printed, it is essential that standard safety and quality measures are followed for 3D-printed parts intended for clinical use. However, the same open-source format that allowed for crucial collaborative development also allows for a lack of standardization and quality measures since the same STL file can produce varying functionalities if printer settings and g-codes differ [37]. To combat this during production, ASTM International enabled public access to their standards [26]. Additionally, the application of a polyurethane finish such as Shellac has been recommended as an additional seal [35,37], thereby preventing filament moisture retention and bacterial and viral growth. Post-processing steps such as these are required for FDM-printed parts because they have rough surface finishes and other inherent defects that are problematic in a clinical setting, since surface roughness promotes biofilm formation [38,39]. Rough surfaces have a greater surface area for adhesion, can shelter bacteria from shear forces during initial adhesion, and are difficult to clean [40]. The rough surface finish and other defects inherent in FDM-printed parts are caused by start–stop errors during the printing process, the ridging effect caused by small bulges that form when printing on a flat surface, the staircase effect that occurs when printing curved surfaces in a layer-by-layer fashion, and warping caused by temperature differences between the print nozzle, newly deposited material, and previously printed layers [41]. Historically, poor surface finish and other defects were tolerable because the printed parts were non-functional. However, technological advances as well as societal needs (e.g., the COVID-19 pandemic) have led to FDM being used to manufacture functional parts and products. Consequently, several strategies have been employed to combat these defects in FDM-printed parts, including: to reduce the layer thickness (at the expense of increased build time and cost), to optimize the build orientation in the X and Y planes, and to use advanced equipment setups with closed, controlled build environments [42]. However, most small desktop FDM 3D printers do not have the functionality to implement many of these strategies. Furthermore, even the most advanced FDM 3D printers cannot produce a smooth surface finish and post-processing is still required. Mechanical and chemical post-processing steps have both been used successfully [43]. For instance, ABS can be smoothed by acetone vapor. Unfortunately, this added step to achieve a smooth surface finish lengthens the manufacturing process and is another obstacle to mass-producing 3D-printed PPE.

### 2.3. Additive versus Conventional Manufacturing Technologies

In general, additive manufacturing technologies cannot match the production volume of some conventional manufacturing methods, but they are important manufacturing tools, nonetheless. Conventional methods, such as injection molding, can produce tens of thousands of units per day with smooth surface finishes that require little-to-no post-processing. A study investigating the FDM print times for face shields and face masks found the average face shield design took 2.5 h just to print, and the average face mask design required about 5 h [27]. Some designs clustered multiple face shields together in a single print and could produce eight face shields in an hour. Even with such designs, a back-of-the-envelope calculation would show that hundreds of printers operating 24 h a day without interruption would be needed to match injection-molding production volume. As discussed earlier, there can be high variability in part quality amongst printers depending on the print settings. Still, 3D printing has been a powerful tool in the fight against PPE shortages, and the 3D printing community was able to rapidly respond to successfully deploy products to those in need. Specifically, 3D printers are faster to set up and can accommodate frequent design changes. They are also better suited for complex designs and customization. Furthermore, small batch productions (between 100 and 1000 units) can be more cost-effective than injection molding due to the required money and time needed for tooling and to optimize the machine operating parameters [44]. Clearly, there will continue to be a need for 3D printing solutions as the world’s economies continue to reopen and supply chains remain fragmented. However, there are challenges to standardization when several different brands and models of FDM machines as well as print materials are used.

### 2.4. Sterilization Considerations

Because most are meant to be reusable, materials must be able to survive multiple rounds of sterilization and/or disinfection to be reused safely. Several sterilization techniques are used by medical facilities including heat sterilization, ionizing radiation, ethylene oxide gas, and hydrogen peroxide gas plasma sterilization [45]. However, many of the thermoplastics used in FDM printers are not compatible with some of these sterilization and disinfecting approaches. For instance, ABS and PLA both have glass transition temperatures below or near temperatures used for heat sterilization (i.e., 75–120 °C) [46,47,48], which can alter material properties and cause shape loss. Household disinfecting and cleaning solutions were shown to effectively re-sanitize PP following E. coli inoculation, while bacterial growth was observed on PLA [49]. However, ethanol and chlorine-based solutions have also been shown to compromise the filtration efficiency of PP materials by removing the static charge from the PP fibers [50]. A similar effect was observed following gamma irradiation [51,52]. Furthermore, evidence of ionizing radiation inducing polymer chain scission or cross-linking in the polymeric network and compromising the mechanical properties has been reported for PP [53] as well as ABS [54] after a single sterilization cycle. The performance of PLA has been shown to be unaffected by irradiation doses used for clinical applications (25 kGy) and doses up to 50 kGy [55,56]. Gas-based approaches are often used for materials that cannot tolerate high temperatures, moisture, and ionizing radiation. Ethylene oxide had been commonly used for low-temperature sterilization (54 °C), but the gas must have direct contact with microorganisms on or in the part being sterilized. Additionally, ethylene oxide gas sterilization requires long post-process aeration to remove toxic gas residue. Hydrogen peroxide gas plasma is another low-temperature sterilization approach that has been successfully used for final sterilization of 3D-printed parts such as surgical guides made of ABS [57,58]. For surgical guide applications, as well as customized PPE, dimensional accuracy is extremely important, but few studies have investigated its impact on the mechanical properties [59,60,61,62]. Overall, most studies on the effects of sterilization and decontamination have measured the effects following a single cycle. Long-term compatibility and potential degradation of 3D-printed PPE and other medical equipment following multiple cycles of sterilization must be evaluated to ensure standard safety and quality.

## 3. Detection and Diagnosis

**Figure 2:**
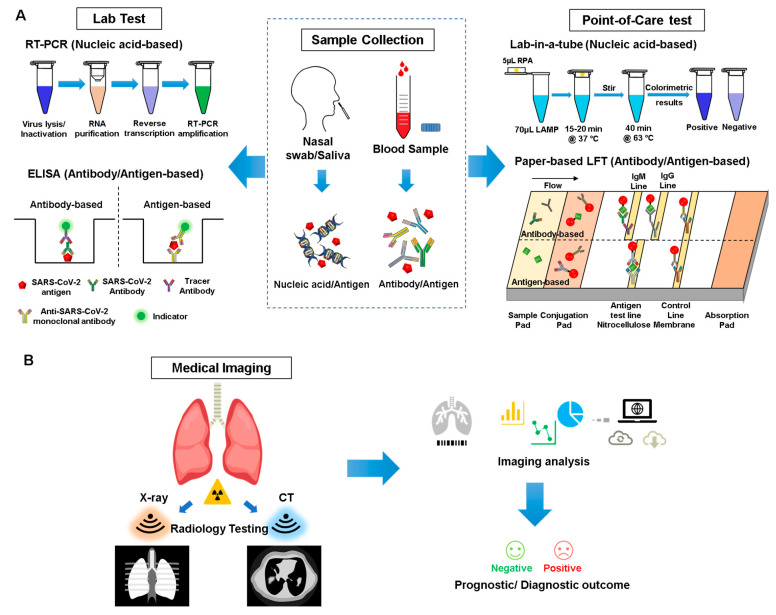
Diagnostic approaches. (**A**) Samples are collected and subjected to various laboratory-based or point-of-care (PoC) tests based on nucleic acid detection, antibody detection, or antigen detection. RT-PCR: reverse transcription polymerase chain reaction; ELISA: enzyme-linked immunosorbent assay; LAMP: loop-mediated isothermal amplification. (**B**) Hospital-based medical imaging can be utilized for disease diagnosis.

Multiple general diagnostic approaches have been implemented for COVID-19, from the molecular level to organ level, including laboratory-based high-throughput screening, point-of-care (PoC) tests, and imaging-based examination (Figure 2). Each diagnostic method has its unique advantages and limitations, and the application of different assays should be performed to ensure a more accurate diagnosis (Table 1).

### 3.1. Sampling/Serology Analysis

The gold standard for diagnosis consists of real-time polymerase chain reaction (RT-PCR) tests to confirm the presence of the virus in an individual (Figure 2). To acquire the necessary genetic data, nasopharyngeal swab tests have been primarily utilized to collect the samples, which are then evaluated using PCR [63]. The Centers for Disease Control has also authorized the usage of oropharyngeal swab tests since they can be administered more easily and are accessible, but reports have found that they are less accurate than nasopharyngeal tests [64]. 

PCR tests have been shown to be highly accurate, and although false positives are rare, false negatives have been more prevalent. It has been suggested in areas with high cases or transmission rate that a secondary test after a negative should be taken, especially if an individual has symptoms [64]. Nasopharyngeal swabs have been mainly used for these tests, and the material must be kept refrigerated if the samples are to be processed in less than 5 days according to the World Health Organization’s guidelines [63]. 

Another diagnostic test that has recently been found to be crucial in fighting this pandemic are serology tests since they can present a more accurate representation of death rates of the disease in nations with widespread COVID-19 infection; furthermore, they indicate those who have recovered from COVID-19 and thus can potentially donate their blood plasma to those still battling SARS-CoV-2 [65]. Although antibody tests are less crucial in detecting active cases, they are important in understanding the spread of the disease. High-throughput assays such as enzyme-linked immunosorbent assays (ELISA) have been used to detect complement antibodies to the SARS-CoV-2 virus (Figure 2).

**Table 1:** Various molecular-level diagnostic techniques.

Lab Test					
Technology	Sample	Target of Detection	Time Cost	Advantage	Disadvantage
RT-PCR	Nasal swab, tracheal aspirate, bronchoalveolar lavage specimens	Viral gene	2–8 h	Gold standard High accuracy	Time consuming Labor intensive High cost Unavailable for remote settings
ELISA	Blood/serum	Antibody	1–5 h	Relatively cheap High throughput	Time-dependent Possible cross-reactivity Not applicable for early diagnosis
	Serum, Nasopharyngeal swabs	Antigen			Limited data available Under development
**Point-of-Care**					
**Technology** **Platform**	**Sample**	**Target of Detection**	**Time Cost**	**Advantage**	**Disadvantage**
LFA Paper-based strip [66]	Blood	Antibody	5–20 min	Rapid Low cost Easy fabrication, modification, and functionalization Simple operation User-friendly Massive production	Low specificity and sensitivity
	Nasal or nasopharyngeal swabs	Antigen			
LAMP Lab-in-a-tube [67]	Nasal swab	Viral gene	30 min	Minimal sample processing No thermal cycling is needed Naked-eye detectable results	Relatively lower accuracy compared to PCR Relative complexity of device compared to rapid antigen tests
LAMP Silicon microfluidics chip [68]				Highly stable No auto-fluorescence Efficient manufacturing	
Electrochemistry Graphene field-effect transistor [69]	Nasopharyngeal swabs [70]	SARS-CoV-2 spike protein	2 min	Rapid Real-time Label-free Unprecedented sensitivity and chemical stability Limit of detection down to 0.2 pM	A more complex fabrication process and dedicated nanotech facilities are required

Serology tests have been shown to be able to accurately predict if a person has contracted the COVID-19 virus, and the main three high-throughput tests that have been used in this pandemic are lateral flow assays, ELISA assays, and chemiluminescent assays (Table 2). Serology tests have also been important in studies that are assessing the role of antibodies in neutralizing the virus [71]. According to a recent 2020 analysis, chemiluminescent assays are far more accurate than lateral flow and ELISA assays [72]. Chemiluminescent assays work primarily by using chemical probes to bind to antibodies, and then with a chemiluminescent reaction the machine can detect a fluorescent signal. ELISA assays work similarly but use enzymes to bind the antibodies, and a color signal may be detected with a follow-up reaction with horseradish peroxidase or other chromatographic reaction-causing agents [73]. Lateral flow assays work similarly to ELISAs; however, they depend on capillary flow to produce chromatographic results. The specificity of the chemiluminescent assay may be the reason why in general they lead to better results while lateral flow may vary since a lot of them are point-of-care and focus on accessibility rather than high throughput. An important consideration is that although the specificity of serological tests appears to be high (Table 2), this is highly dependent on the day of sampling after infection.

### 3.2. Point-of-Care Devices for Diagnosis

Although RT-PCR is the gold standard of diagnosis of COVID-19, it has some limitations such as being time-consuming (i.e., typically taking 90–120 min), labor-intensive (well-trained personnel and a lab environment are needed), and relatively expensive [84]. With the rapid spread of this pandemic, a rapid, sensitive, inexpensive, and user-friendly diagnostic tool is urgently needed. Point-of-care (PoC) devices for rapid diagnosis of infectious diseases have been receiving greater attention in recent years. PoC devices have many advantages compared to conventional lab-based technologies, including being affordable, sensitive, specific, user-friendly, rapid and robust, equipment-free, and deliverable (ASSURED) to end users [85]. With PoC devices, the test results can be obtained and delivered to patients in a more rapid manner, instead of using complex and expensive lab settings. Moreover, these devices enable rapid medical decision making and reduce the risk of human-to-human transmission. With all of these advantages, PoC devices have the potential to meet the urgent need for rapid diagnosis of COVID-19.

Based on the targets, PoC devices can be divided into three major types: nucleic-acid-based, antibody-based, and antigen-based tests (Figure 2). In nucleic-acid-based tests, patient’s sputum and nasal secretions are typically used as samples, and direct detection of the genetic material of SARS-CoV-2 will be performed [84]. To meet the requirements of being PoC tests, RT-PCR is obviously not recommended due to its time-consuming and labor-intensive properties; therefore, a loop-mediated isothermal amplification (LAMP) is the more preferred method to be employed in PoC devices. Compared to RT-PCR, LAMP takes a much shorter time (i.e., it can be completed within 30 min), and more importantly, it can be performed under a constant temperature [84]. These two properties make LAMP a proper testing technology for PoC devices. A recent study developed a lab-in-a-tube system called “Penn-RAMP” for diagnosis of COVID-19 [67]. It consists of two isothermal amplification processes, a recombinase polymerase amplification (RPA) at 38 °C, and a LAMP at 63 °C to achieve a higher sensitivity (i.e., 10× higher compared to RT-PCR or LAMP only with purified targets and even 100× higher sensitivity with rapidly prepared samples). The entire process can be performed in a single tube, and a colorimetric detection by the naked eye works as a readout, making it a proper candidate for a nucleic-acid-based PoC diagnostic tool for COVID-19.

On the other hand, in antibody-based tests, the sample typically consists of the patient’s blood [66]. In contrast to nucleic-acid-based tests, this method can detect SARS-CoV-2 in an indirect way by targeting the antibodies (typically IgG/IgM) against the virus. Previous studies have shown that IgG/IgM will be produced to fight the virus after infection of SARS, and IgM can be detected after 3 to 6 days, while IgG can be detected in a patient’s blood after 8 days [86]. Since SARS-CoV-2 belongs to the same family of viruses which cause SARS and MERS, the production process of IgG/IgM should be similar and therefore can be applied as a potential diagnostic tool for COVID-19. Currently, there are several commercialized PoC devices based on detecting antibodies against SARS-CoV-2; in particular, most of them are IgG/IgM lateral flow test strips. These antibody-based tests offer a major advantage compared to nucleic-acid-based tests in that they exhibit shorter reaction times, providing faster results (typically 5–20 min). Similar to nucleic-acid-based PoC tests, these devices are easy to use and able to deliver “naked-eye” test results. However, it is important to note that since antibody levels can vary during the infection process, these tests should not be used solely as a decisive diagnostic tool for COVID-19 due to its time-dependent property. For example, these tests may provide a false negative result, especially at the initial stage of infection, when there is only a low level of antibodies present in a patient’s blood, which may be undetectable by the device. Thus, combining both nucleic-acid-based and antibody-based test results to deliver a more accurate diagnosis may be a better option. In addition, with the spread of vaccine administration, antibody-based testing may not work as a good criterion for the diagnosis of SARS-CoV-2. A SARS-CoV-2 antibody test result can be positive due to a SARS-CoV-2 infection or in response to antibodies from a COVID-19 vaccine. With the development of monoclonal antibodies specifically binding to the SARS-CoV-2 antigens, an increasing amount of research focus has been turned from antibody-based tests to antigen-based tests, with direct detection of the viral antigen to indicate an active viral infection.

Based on the fabrication process, PoC devices can be divided into several groups, of which two major ones are chip-based and paper-based biosensors. Chip-based biosensors are typically made of polydimethylsiloxane (PDMS) due to its transparency, biocompatibility, and cost-effectiveness [87]. However, due to the porosity and gas permeability nature of PDMS, cross-contamination may occur as reagents in the PDMS chamber may evaporate while performing the nucleic acid amplification within a PDMS-glass biosensor. To overcome this and with the help of a laser ablation fabrication process, polymethyl methacrylate (PMMA) is a proper alternative due to its low evaporation rate and low non-specific nucleic acid adsorption [88]. All nucleic-acid-based, antigen-based, and antibody-based tests can be achieved with chip-based biosensors [89,90]. It has some unique advantages such as allowing for precise manipulation of a small amount of fluidic sample and high-throughput capability, while at the same time exhibiting some limitations, for instance, a complex fabrication process, e.g., photolithography, deposition, and etching, which typically requires a clean room process [91]. Several chip-based biosensors specific to COVID-19 diagnosis have been published recently. For example, based on RT-LAMP and fluorescence, Ganguli et al. developed a rapid isothermal amplification and portable detection system for SARS-CoV-2 [92]. The system is based on an additively manufactured 3D cartridge and a smartphone-based optical reader. To conduct the test, the nasal sample is acquired from the patient with a nasopharyngeal swab and transported in a viral transport medium (VTM) solution to transfer the viruses. Afterwards, the VTM aliquots are thermally lysed at 95 °C for one minute and then loaded into the microfluidic cartridge simultaneously with the RT-LAMP reagents via syringes. Finally, the nucleic acid amplification with intercalating fluorescent dye is conducted at 65 °C by putting the cartridge into the smartphone cradle. A smartphone camera is used to record the fluorescent signal in real-time followed by image analysis with ImageJ. The limit of detection (LOD) of this detection system is 50 copies of RNA per μL, and the whole process can be finished within 40 min. The sensitivity and specificity of this detection system were 100% as determined by testing 20 clinical confirmed samples. 

Compared to chip-based biosensors, paper-based biosensors are cheaper, easier to use, and have a simpler fabrication process, which allows for mass production [93]. Typical fabrication techniques used for paper-based biosensors include wax printing, alkyl ketene dimer (AKD) printing, flexographic printing, and layer-by-layer 3D affixing [94]. The aforementioned IgG/IgM lateral flow test strip is a good example of a paper-based biosensor. Although nucleic-acid-based [95] or antibody-based tests can be performed with paper-based biosensors, most commercial products are antibody-based since nucleic-acid-based tests require extra steps (e.g., RNA extraction, LAMP). An IgG/IgM lateral flow test strip typically consists of a sample pad for sample loading; a conjugation pad sprayed with gold COVID-19 conjugate (i.e., a surface antigen from SARS-CoV-2 conjugated to colloidal gold nanoparticles) together with gold rabbit IgG conjugate for control; a nitrocellulose membrane consisting of an M Line coated with anti-human IgM antibody, a G Line coated with anti-human IgG antibody, and a control line coated with anti-rabbit IgG antibody; and an absorbent pad for waste absorption [66]. This biosensor will deliver a visible readout on test lines, and with combined data of IgG and IgM results, it can help detect patients at different infection stages. Moreover, it has a higher sensitivity than either IgG-only or IgM-only tests. However, one of its major limitations is that it is a qualitative assay rather than a quantitative one. Based on the same mechanism, an antigen-based lateral flow test strip can be developed by simple modification of the gold nanoparticle conjugate and antibodies on the test line. To achieve this, a primary anti-SARS-CoV-2 specific monoclonal antibody is conjugated to the gold nanoparticles instead of antigen. At the same time, a secondary anti-SARS-CoV-2 capture antibody for the same antigen is immobilized on the nitrocellulose membrane as the test line to generate a visible readout [96]. A fluorescence readout can be achieved by replacing the gold nanoparticle–antibody conjugate with a fluorescence-labeled detection antibody [97].

Besides chip-based and paper-based biosensors, many other types of biosensors have been developed, typically based on nanomaterials, including nanowires, nanotubes, and nanoparticles, due to their electrochemical and mechanical compliance, optical transparency, bendability, and high electrical conductivity [90,98]. Two-dimensional materials such as graphene play an important role in this field benefiting from their high charge mobility and surface area, offering high performance and low-cost biosensing ability. For example, Torrente-Rodríguez et al. developed a graphene-based, electrochemical-based multiplexed telemedicine platform, the SARS-CoV-2 RapidPlex, for COVID-19 diagnosis and monitoring [99]. This detection system consists of four working graphene electrodes, an Ag/AgCl reference electrode, and a graphene counter electrode, patterned on polyimide substrate via CO_2_ laser engraving and functionalized with proper receptors, enabling simultaneous quantification of different biomarkers regarding COVID-19 (e.g., nucleocapsid protein, anti-spike protein IgG and IgM, and C-reactive protein) through sandwich- and indirect-based immunosensing strategies, either with patient serum or saliva sample. This multiplexing feature helps provide more informative results, including viral infection, immune response, and disease severity of COVID-19. In addition, this platform offers beneficial features such as low cost (the material cost of the unmodified RapidPlex platform is within USD 0.05, and the additional chemical reagents cost is at the level of dollars) and short sample-to-answer time (target capture can be as low as 1 min).

As PoC devices offer several advantages, such as being cost-effective, easy-to-use, and timesaving, the application of such devices will be able to address the urgent need for a rapid diagnostic tool for COVID-19 under the circumstance of a global pandemic. However, compared to the gold standard RT-PCR, the available commercial PoC devices have a relatively lower clinical sensitivity and specificity. Therefore, further investigation on methods to improve sensitivity will enhance the reliability and application of PoC devices for rapid diagnosis.

### 3.3. CRISPR/Cas Technology

Clustered regularly interspaced short palindromic repeats (CRISPR) and specific high-sensitivity enzymatic reporter unlocking (SHERLOCK) display promise sensitive, specific, and reliable assays for screening and diagnosing cases of SARS-CoV-2. These techniques offer the advantages of (1) increased accessibility through decreased dependence on RT-PCR equipment (RT-PCR is the current standard employed by the EUA-approved assay developed by the Centers for Disease Control and Prevention [100]), (2) increased speed of assay compared to RT-PCR, which typically requires a greater than 24 h turnaround time due to the need to ship samples to equipped laboratories [101], and (3) increased availability to bolster current efforts to reduce disease spread.

Multiple research groups have begun development on CRISPR- and SHERLOCK-based mechanisms for PoC, rapid, and sensitive detection of SARS-CoV-2. The primary goal of these strategies is to simplify and refine the CRISPR process to allow for quick and simple processing of potential disease samples. A detection method termed SARS-CoV-2 DNA endonuclease-targeted CRISPR trans reporter (DETECTR) has been developed, which utilizes simultaneous reverse transcription and isothermal amplification on nasopharyngeal and oropharyngeal RNA samples followed by Cas12 detection of envelope and nucleoprotein sequences [101]. This DETECTR assay can be visualized on a lateral flow strip and run in approximately 30 to 40 min with a limit of detection of 10 copies per µL [101]. Another detection method termed all-in-one dual CRISPR-Cas12a (AOID-CRISPR) utilizes a pair of Cas12a–crRNA complexes to improve the sensitivity and specificity of the CRISPR-based assay [102]. This system involves recombinase polymerase amplification (RPA) to allow for strand displacement and binding. AOID-CRISPR can output results with a limit of detection of 1.3 copies per µL in 40 min [102] in comparison to other assays, which have shown limits of detections of 100 copies per µL in 50 min [103] and 10 copies per µL in 2 h [104]. Of note, the sensitivity of all of these assays is comparable to RT-PCR assays, which have a limit of detection of approximately one copy per µL. Some studies further claim that the water baths necessary for CRISPR assays can be controlled utilizing only a commercially available low-cost sous-vide cooker, providing a significant decrease in cost relative to RT-PCR [103]. Moreover, a clinical study of patients at Siriraj Hospital in Thailand found that of 154 nasopharyngeal and throat swabs, SHERLOCK assays with a fluorescence readout were 100% specific and 100% sensitive and SHERLOCK with a lateral-flow readout was 100% specific and 97% sensitive, results that were echoed in full viral load clinical samples and pre-operative samples [105]. Altogether, these studies highlight how CRISPR- and SHERLOCK-based assay mechanisms are promising techniques for the detection of SARS-CoV-2 due to their decreased cost, improved ease of access, and increased speed of results. However, further research must be conducted to assess the viability of CRISPR- and SHERLOCK-based PoC devices, where considerations should include the sensitivity and specificity of these assays in regard to cost and ease of access. Ideally, CRISPR- and SHERLOCK-based assays would be able to be utilized in a handheld single cartridge format while not losing any sensitivity compared to traditional serological assays. 

### 3.4. Medical Imaging and AI Assistance

Medical imaging approaches such as X-ray and computed tomography (CT) played an essential role in the global fight against COVID-19, and recently emerging artificial intelligence (AI) technologies can strengthen the power of these imaging tools to help medical specialists [106]. Although RT-PCR tests serve as the gold standard for confirming COVID-19 patients [107], this assay has proven to be inadequate in many areas that have been severely hit, especially during early outbreak of this disease. Due to several factors, such as sample preservation and quality control, the sensitivity of RT-PCR in laboratory tests has been reported to be insufficient [108,109]. Therefore, based on clinical practice, easily accessible imaging approaches such as chest X-ray and thoracic CT can serve as an alternative, providing valuable information and assistance to clinicians [110,111] (Figure 2).

Imaging approaches can be utilized to not only take images of the chest but also the abdominal region, cranial cavity, and other areas of the body that can be affected by COVID-19, providing more information, compared to other approaches, to make a more precise diagnosis [110,112,113]. More importantly, X-ray and CT systems are equipped with cameras for patient monitoring purposes [106]. During the COVID-19 outbreak, these cameras provided opportunities to establish a contactless scanning workflow. This enabled patient monitoring in real time from the control room via a live video stream from the camera. However, due to infection control issues related to patient transportation to CT suites, inefficient CT room decontamination still poses a challenge for technicians and residents to avoid becoming infected [114]. In addition, from only the overhead view of the camera, scanning parameters such as scan range make it difficult to make a diagnosis. In such cases, AI may serve as a powerful tool that can be potentially utilized to better diagnose, predict, and treat infectious diseases, such as COVID-19 [115]. 

## 4. Disease Modeling and Drug Discovery

**Figure 3:**
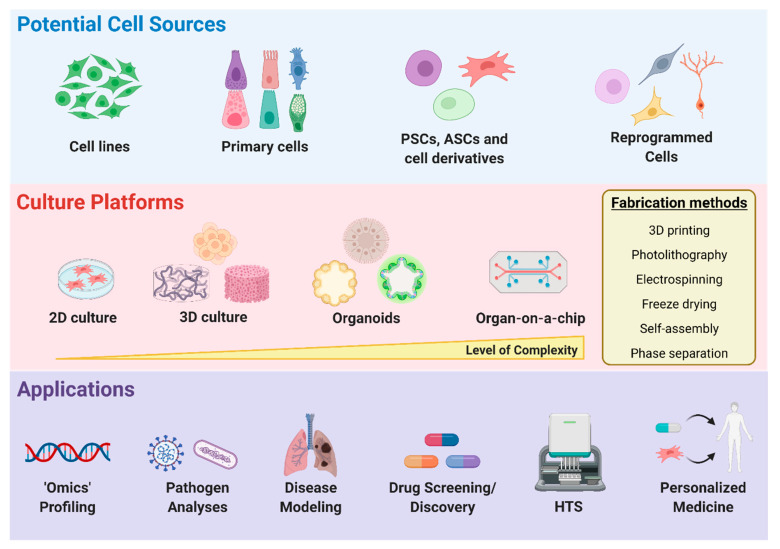
Disease modeling and drug discovery systems for COVID-19. Various cell sources can be implemented for disease modeling, including cell lines, primary cells, pluripotent stem cells (PSCs), adult stem cells (ASCs), stem cell derivatives, and reprogrammed cells. Cells can be cultured in platforms ranging from 2D surfaces to more complex organ-on-a-chip devices and are highly valuable for a broad range of biomedical and regenerative medicine applications.

In order to develop potent therapeutics against infectious diseases, such as COVID-19, it is important to utilize a model that can accurately depict the behavior of the virus and the pathology of the disease. Cells from various sources can be implemented to not only study the replication and infection kinetics of the virus but also the effects of SARS-CoV-2 infection on specific human tissues (Figure 3). Although cells have been typically cultured on 2D surfaces, there is a piqued interest towards growing cells in 3D microenvironments. With an expanding selection of biomaterials and significant developments in fabrication methods, including 3D bioprinting, the creation of 3D architecture that mimics the in vivo microenvironment and allows for long-term cell culture has become more feasible [116,117,118], providing great promise for disease modeling, tissue engineering, and regenerative medicine. Moreover, advancements in organ-on-a-chip platforms provide a new mechanism to study human disease pathology and allow for the creation of more targeted therapeutics. 

### 4.1. Cell Sources for Disease Modeling

Several immortalized cell lines such as Vero E6, Huh7, and HEK293T cells have been utilized to investigate mechanisms of SARS-CoV-2 infection [12,119,120], in addition to providing a way to amplify and propagate the virus. Although immortalized cell lines do not mimic human physiological conditions and are not representative of all cell types, these cells, which can proliferate indefinitely, are inexpensive to grow and maintain and easy to genetically manipulate. For instance, overexpression of ACE2 receptor and TMPRSS2 in these cell lines can be performed to study SARS-CoV-2 infection and improve viral titers, respectively [121]. 

In cases where aspects of native physiological function need to be maintained, primary cells, stem cells, and reprogrammed cells can serve as alternative sources. Primary cells can be isolated from specific tissues, potentially providing fully differentiated cell types that may be better predictors of what is occurring in vivo. For example, primary human airway epithelial cells, which can serve as an in vitro physiological model of human lung origin, have been utilized to show the cytopathic effects and morphogenesis of SARS-CoV-2 infection [1,122]. However, primary cells are expensive, heterogeneous, highly variable depending on the batch source, not easily accessible for specific cell types, and require the sacrifice of animals or donor tissue availability for cell isolation. On the other hand, stem cells, including embryonic stem cells, induced pluripotent stem cells (iPSCs), and adult stem cells (ASC), can self-renew, thereby yielding large quantities of undifferentiated cells that have potential to differentiate into all cell types, depending on their potency, making them highly valuable for regenerative cell therapy, disease modeling, and drug discovery [123,124,125]. More importantly, their derivatives display native function characteristics, an important aspect when creating models to investigate how SARS-CoV-2 may infect tissues in vivo. Indeed, a recent study demonstrated that iPSC-derived cardiomyocytes are susceptible to SARS-CoV-2 infection and can be utilized as a platform to study the mechanisms by which SARS-CoV-2 infects cardiomyocytes in vitro [126]. Furthermore, in a separate study where distinct hPSC derivatives were infected with a SARS-CoV-2 pseudo-entry virus and luciferase activity was used as a readout of viral infection efficiency, it was determined that pancreatic endocrine cells, cardiomyocytes, and dopaminergic neurons were permissive to SARS-CoV-2 viral entry, whereas endothelial cells, microglia, macrophages, and cortical neurons had relatively minimal to no infection [127]. However, existing challenges such as high cost, length of the differentiation process, purity of the derived population, and limited maturation capability are important factors to consider when utilizing stem cells as an initial cell source [128]. 

One way to overcome some of these drawbacks may be to use cells derived through reprogramming strategies using exogenous transcription factors, small molecule compounds, and biomaterials [129,130,131,132,133]. In particular, trans-differentiation or direct reprogramming has been applied to derive a broad range of cell types that display more mature phenotypes and can be obtained on a shorter time scale, yielding advantageous properties that are beneficial for disease modeling and drug screening applications. In addition, directly reprogrammed cells retain age-related characteristics [134,135], compared to iPSC reprogrammed cells, suggesting these cells may be more applicable for examining the effects of aging and COVID-19, which is of particular importance as COVID-19 disproportionately affects elderly populations. Low efficiencies of target cells are still a major issue with direct reprogramming strategies; therefore, purification of derived populations should be performed, especially when these cells will be used for specific downstream applications, such as high-throughput screening. 

### 4.2. Two-Dimensional vs. Three-Dimensional Microenvironments

All the aforementioned various cell sources are routinely cultured as monolayers in two-dimensional (2D) cell culture platforms. However, a 2D culture is unable to capture the complex 3D microenvironment cells typically experience in vivo, and emerging evidence indicates that cell behavior and phenotype can differ between 2D and 3D environments [136,137]. As a result, there have been substantial improvements to 3D cell culture systems, as it is becoming more widely accepted to implement these systems for disease modeling and drug discovery [116,138]. A simple 3D culture system is to form spheroids through cell aggregation using the hanging drop method, non-adherent spheroid molds, bioreactors, magnetic molds, or low attachment culture plates [116,139,140]. Still, these cultures are associated with long maturation times, limited size and shape reproducibility, and lack cell–matrix interactions, which are important when recreating tissue architecture. As such, spheroids or single cells can be embedded within or grown on hydrogel biomaterials that are composed of synthetic or naturally derived polymers that provide a 3D network [116,141]. Natural biopolymers, including collagen, gelatin, hyaluronic acid, and alginate, exhibit similar biochemical properties as the natural extracellular matrix (ECM), providing improved biocompatibility and pre-existing cues that can regulate cell behavior. On the other hand, synthetic hydrogels can be designed to mimic natural ECM properties and are chemically and physically well defined, thereby overcoming certain disadvantages associated with natural polymers, such as batch variability, limited reproducibility, and difficulty in purification. More importantly, the biochemical and physical parameters, such as stiffness, porosity, cell-adhesive ligands, and viscoelasticity of these 3D synthetic scaffolds, can be further tailored to control the tissue microenvironment, in addition to cell fate and function [116,142]. Therefore, the most appropriate 3D cell culture model should be selected depending on the specific application. Various fabrication methods, including but not limited to electrospinning, freeze drying, phase separation, self-assembly, bioprinting, and photolithography, can be utilized to engineer 3D biomimetic scaffolds using a “top-down” or “bottom up” approach [143,144,145,146]. Although some studies have utilized 2D monolayers to investigate cellular susceptibility to SARS-CoV-2 infection [126,127], the development of an alveolosphere culture system [147], which allows for the propagation and differentiation of human alveolar type 2 cells, and organoids have been implemented as 3D models of SARS-CoV-2 infection. 

#### Organoids

Organoids are another remarkable 3D culture system that can be utilized to study human development and disorders [148,149], such as infectious diseases and cancer. Although organoids can be generated from patient biopsy samples, most are commonly established from PSCs or ASCs, whereby cells undergo a self-organization process that mimics human development in vitro and allows for the generation of complex 3D structures whose architecture and functionality are similar to in vivo organs. Organoids can be cultured in engineered matrices to improve organoid generation, reproducibility, and maturation [150]. Moreover, organoid-forming stem cells and organoids can be combined with 3D bioprinting to generate macroscale tissues with self-organized features [151]. As organoids are composed of organ-specific cell types and can recapitulate human organ physiology, these systems have been highly valuable as a disease modeling and virology platform for COVID-19 research. Thus far, human bronchial [152], lung [153], kidney [154], liver [155], intestinal [156,157,158], whole eye [159], and brain [160,161,162,163,164,165] organoids have been generated and implemented to study the tissue tropism of SARS-CoV-2. Though organoids provide an opportunity to study human disease and can complement existing animal models, organoid technology is still in the nascent developmental stages and some challenges remain to be addressed. For instance, performing investigations at a whole organ level can be challenging, and there is currently no standardization of protocols and procedures, potentially leading to heterogeneous results among different research groups. In addition, some cellular components may be lacking in organoid models, for example immune cells. Further development of co-culture systems with organoids and the incorporation of immune cells within organoids would be the ideal 3D platform for the study of immunological responses to pathogens, such as SARS-CoV-2. Moreover, genomic, transcriptomic, and cytokine profiling of these immune co-culture models can be performed to further reveal cytokines and signaling pathways involved during SARS-CoV-2 infection, providing a platform that can be utilized to not only study the mechanism of pathogenesis but also for the potential identification of immunomodulatory drug candidates.

### 4.3. Organ-on-a-Chip

Although animal models contribute to our understanding of the mechanisms of lung disease, animals and humans are substantially different, revealing the existing controversy of the validity of in vivo research. For example, animals do not necessarily develop pathologies such as asthma or tuberculosis similar to human beings, which may account for more than 80% of drugs that pass the pre-clinical animal testing stage in mice but fail in clinical trials [166,167]. As such, in vitro models can allow for the study of airway and parenchyma in the lung by providing tissue architecture and a functional microenvironment. In vitro models range in their complexity and accuracy in rebuilding different aspects of the lung, from the simplest 2D model for monolayer cell culture to 3D in vitro models mimicking the architecture and functional features of lung, wherein advanced 3D in vitro models include an air–liquid interface to simulate airway and parenchyma mechanics of the lung [166]. Considering the microfluidic features of the human lung, organ-on-a-chip devices allow for modeling of pathological conditions of the lung.

**Figure 4:**
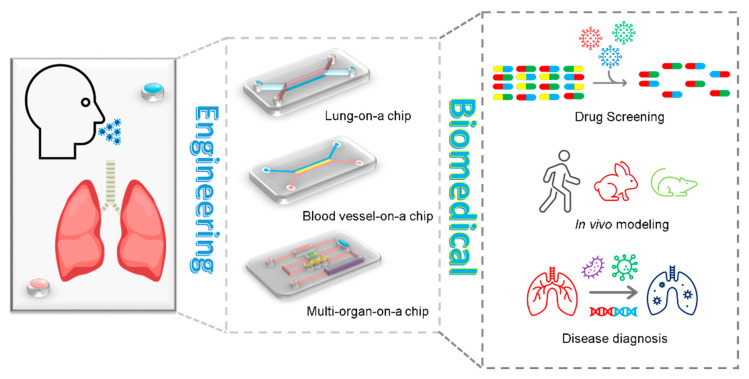
Organ-on-a-chip systems for COVID-19. Organ-on-a-chip devices can be engineered to study human disease pathology, expedite drug screening, and for the development of novel targeted therapeutics.

Organ-on-a-chip combines technological advances in microfluidics, cell engineering, and simulation microenvironment of target cells, enabling the fabrication of a customized cellular microenvironment with precise mechanical, structural, and biochemical control [168]. Organ-on-a-chip technology reveals features of a specific tissue microenvironment and architecture within a microfabricated device, which can exhibit functional hallmarks of native tissue and powerfully predict in vivo events by an in vitro model [169]. During the breakout of COVID-19, organ-on-a-chip devices could be utilized to not only mimic airway conditions to analyze disease pathogenesis but also expedite drug screening and identify potential candidates against pandemic viruses [170,171] (Figure 4). In particular, lung-on-a-chip (LOC) devices incorporate an alveolar membrane for epithelial and endothelial cell seeding and can undergo mechanical stretch with mechanical actuation of the diaphragm-inspired lower membrane by an electro-pneumatic pump. Using LOC devices where human lung airway epithelium was cultured on an air–liquid interface membrane and fed by continuous medium flow, the whole process of COVID-19 infection including viral entry, replication, strain-dependent virulence, host cytokine production, and recruitment of circulating immune cells (e.g., neutrophil) in response to virus infection could be investigated, while at the same time enabling the screening of existing FDA-approved drugs [170]. In this study, an LOC device demonstrated that amodiaquine was effective against pseudotyped-CoV-2 virus in human organ chips and also prevented infection and transmission of native SARS-CoV-2 in hamsters [170].

To fabricate an organ-on-a-chip device, the most commonly used microfabrication technology platform is soft lithography. Different from solid-state integrated circuits, in soft lithography there is no etching or deposition involved in the majority of the fabrication process [172]. More importantly, soft-lithography-fabricated chips are highly compatible with various materials, such as PDMS and polyurethane elastomers [169], providing an opportunity to fabricate different chips that can mimic the mechanics of different organs such as lung-on-a-chip [173], liver-on-a-chip [174], and gut-on-a-chip [175]. However, soft lithography is a multi-step process requiring masks, dedicated equipment, and expertise with the technique [176]. In addition, soft lithography can only be applied to one side of the device while the other side remains flat [177]. 

To address these shortages, 3D and 4D printing can be utilized for organ-on-a-chip fabrication. Three-dimensional printing can provide good manufacturing practice for the fabrication of highly complex devices. The layer-by-layer plus one-step continuous printing technique is capable of producing various complex 3D structures of organ chips [178,179]. Moreover, these 3D-printed multilayered devices can be composed of multiple materials to mimic various organs or different sections of an organ and analyze different data using one chip with multi-sensors [180,181]. Yet, 3D printing of organ-on-a-chip has several remaining challenges, such as the materials for printing organ chips having a narrow range and biocompatible resolution and cryopreservation of printed tissues requiring further consideration [169]. Additionally, based on rapid freeze prototyping (RFP), 4D bioprinting processes have allowed for the development of new nano-functionalized chips, which are highly flexible and customizable and allow for the control of macro and micro porosity in a biological scaffold using RFP technology, enabling its shape memory in the rehydration process [182]. As a result, the 4D bioprinted construct can be transformed into a new and more complex shape when it is subjected to an environmental stimulus [182].

Besides the inner structure of organ chips, auxiliary equipment such as valve control and on-line monitoring are also important parameters in chip platforms. The valve tends to be smaller and smarter on chips to save more space and control the flow of specific conditions. For example, in a point-of-care medical diagnostic chip, a sliding strip acts as a valve to control the serial steps of sample addition and interaction [183], and a smart sensor acts as a check valve in a nutrient monitoring chip [184]. In addition, for real-time monitoring of conditions within a chip, on-line monitoring techniques have rapidly evolved in the past decade and can be applied to various on-chip fields, such as neurochemical monitoring and organoid behaviors [185,186]. More importantly, on-line monitoring allows for technicians and residents to directly avoid touching samples donated from COVID-19 patients, which is much safer than traditional assays. Although high-throughput devices can potentially enhance the efficiency of drug screening and bio-sample testing, this still remains a challenge for drug screening chips.

### 4.4. High-Throughput Screening

High-throughput screening (HTS) can be utilized for various applications such as disease modeling, compound screening, drug discovery, and genome-wide RNA interference assays. For instance, compounds that can prevent viral infection or replication can be identified through HTS assays [187]. Moreover, this technology can be applied to specific cell types to discover potential candidates that prevent damage or restore cell function after viral infection. These cell-based assays allow for drug combinatorial screens and dose assessments which can be difficult to perform in animal models. As pluripotent stem cells are highly valuable for basic and applied research, efforts have been made to adapt these cells to large-scale culturing conditions required for HTS applications [188]. Similarly, to address the issue of variability that may arise during manual culture, automated liquid-handling platforms have been developed for the high-throughput production, maintenance, and differentiation of iPSCs [189]. However, as aforementioned, 2D cell cultures lack the physiological microenvironment of in vivo tissue, thereby limiting accurate modeling of drug diffusion kinetics and potentially yielding misleading drug cellular responses. As such, significant advancements have been made to large-scale culturing platforms for 3D organoids with ECMs [190,191], providing more physiologically relevant models for high-throughput applications. HTS can be applied to not only perform toxicity screens and identify compounds that can enhance organoid differentiation but also multiplexed with other technologies to allow for automated multidimensional phenotyping of organoids [192,193]. In addition, hydrogels that can be implemented with existing HTS platforms are promising, and further technological advances in 3D bioprinting will potentially allow for the creation of reliable and reproducible 3D cell cultures for HTS [194,195].

For drug repurposing strategies, virtual screens using computer-aided drug screening can be an efficient approach to identify promising drug repurposing candidates for COVID-19 treatment [196,197,198,199,200,201]. The potency and efficacy of potential candidates can be tested and validated through HTS and in vitro studies. Together, structure-assisted drug design and virtual drug screening, in combination with HTS, are highly valuable as they could potentially shorten the time and reduce the cost of drug discovery.

### 4.5. Machine Learning

COVID-19 has thrown the world into a pandemic, and technologies have been utilized to combat and minimize the spread of the virus causing the disease, SARS-CoV-2. Machine learning is one technology that has been useful in combating the virus because of its widespread application to viral detection and drug development [202]. Machine learning has been applied clinically to detect COVID-19 in a CRISPR-based surveillance system, and deep learning has been utilized to analyze CT images to diagnose patients [203]. In addition, when efforts began to identify the SARS-CoV-2 sequence and origins, machine learning allowed for classification of the genome in an easy and scalable manner [204]. This technology has also been used to not only forecast population spread in using various models to predict short-term forecasting but also in combination with epidemiological, clinical, and genetic data to optimize public health measures, patient survivability, and treatments [202]. Moreover, machine learning can be utilized to predict the targeting of vaccines to the whole virus or specific viral proteins (e.g., S-protein, membrane, and N-protein). Overall, machine learning is a useful tool that can be widely applied to address various aspects of pandemic-causing diseases.

## 5. Therapeutics Development

There continues to be an urgent need for therapies to protect the human population against COVID-19. Vaccines are a promising tool to impede the spread of SARS-CoV-2, and there are various types of vaccine platforms (Table 3). Although several companies have successfully demonstrated efficacy and safety of their vaccines, including Pfizer’s Comirnaty which has now received FDA approval, a wide range of therapies are still being evaluated, such as antiviral agents, anti-inflammatory and anti-thrombosis drugs, biomaterial-based therapies, and stem-cell-based therapies [17,205,206], with 6434 ongoing and completed COVID-19 studies listed on the ClinicalTrials.gov website of the World Health Organization as of 13 April 2022 [207]. So far, only a few drugs have demonstrated efficacy against COVID-19 in clinical trials. Here, we provide an updated overview of the promising medications or therapeutics, including antiviral replication and transcription agents, anti-inflammatory drugs, anti-thrombosis, biomaterial-based therapies, and stem-cell-based therapies, for the treatment of COVID-19 disease.

### 5.1. Antiviral, Anti-Inflammatory, and Anti-Thrombosis Therapy

Antiviral drugs involve small-molecule inhibitors intended to block pathways of the viral replication cycle and therefore mitigate viral infection. Some clinical trials have shown that remdesivir was superior to a placebo in shortening the recovery time in hospitalized patients with COVID-19 and had evidence of lower respiratory tract infection [228,229], yet other trials show no efficacy. The lack of definitive results on remdesivir and similar antivirals, as well as reports of unfavorable symptoms, has led to the WHO releasing a conditional recommendation against their use in hospitals, and the FDA has yet to approve an antiviral drug for COVID-19 treatment [230,231].

SARS-CoV-2 usually triggers a hyperinflammatory response in severe patients characterized by excessive cytokine release, often referred to as a “cytokine storm” [232,233]. The cytokine dysregulation and influx of inflammatory myeloid cells can lead to lung infiltration and critical symptoms, including sepsis, shock, respiratory failure, acute respiratory distress syndrome (ARDS), multiorgan system dysfunction, and death [232,233]. Emerging clinical trial data suggest that individual immunomodulatory drugs including inhibitors of IL-1, IL-6, GM-CSF, TNFα, and Janus kinase (JAK) alone and in combination can dampen the hyperactive immune system in severe COVID-19 [234,235,236]. Some pilot studies found that specific human monoclonal antibodies isolated from convalescent COVID-19 patients can effectively inhibit virus infection and alleviate infection-related lung damage [237,238,239,240,241]. Different neutralizing mAbs from convalescent COVID-19 patients can be combined to improve treatment efficacy. However, antibody production is not the most feasible option, as there will be retargeting delays in available treatments with each new virus mutation, as opposed to vaccinations, which are more likely to still be effective [242]. Further challenges include limited sources and high production costs [243,244,245,246].

While ARDS and related symptoms are the primary causes of COVID-19 mortality, studies have indicated that patients may also experience coagulation disorders, such as thrombosis, following infection. Autopsy studies have demonstrated the presence of fibrin thrombi and distension of small blood vessels with extensive fibrin deposition in patients [247]. Continued anticoagulation treatment options include low-molecular-weight heparin, direct oral anticoagulants, fondaparinux, and warfarin. However, it is also important to consider factors such as patient preferences, renal or hepatic function, and bleeding risk when evaluating treatment options. Future study is necessary to identify the clinical manifestation of thrombotic complications caused by SARS-CoV-2 in order to allow for more specific treatment planning and better provide for patient health and wellbeing.

### 5.2. Biomaterial-Based Therapies

Although there is a diverse array of drug candidates for SARS-CoV-2 treatment, many of them are not ideally suited for systemic administration using standard oral or intravenous routes, due to short half-lives or off-target effects. In this regard, engineered biomaterials are promising as diagnostic and therapeutic strategies for COVID-19 via controlled drug delivery, therapeutic immunomodulation, and tissue regeneration [248,249].

Biomaterial-mediated drug delivery tools are expected to play a paramount role in the success of therapeutic approaches [250]. Antiviral nanoparticles (NPs) coated with undecanesulfonic acid (MUS)-containing ligands as long and flexible linkers can mimic heparan sulfate proteoglycans (HSPG) as the highly conserved target of viral attachment ligands [251]. Recent advancements in NP fabrication technology allow viral glycoprotein structures to be synthetically reproduced with great precision and act as a vaccine would, even surpassing typical vaccine performance in some studies [252]. A broader immune response against multiple viral strains may also be solicited by coating NPs with multiple antigens [252]. Just as blood serum from recovered COVID-19 patients can be used as a treatment due to the monoclonal antibodies their bodies produce, NPs coated in these antibodies provide a more controlled and scalable delivery of the therapy. Furthermore, as biological materials have rough surfaces for improved adhesion, biomaterial advancements mean that nanomaterials can be manufactured with a similar spiky shell to prolong contact with the virus [253]. NPs coated in cell membrane decoys have been shown to prolong half-life, improve immunocompatibility, and prevent SARS-CoV-2 infection in several in vitro studies [252]. Nanosized delivery vehicles (for example, peptide, inorganic, lipid, and polymeric NPs) for systemic drug delivery overcome the degradation of therapeutic agents by nucleases, fast clearance, limited cellular uptake, and off-target effects [254,255]. The expectation is that these nano-delivery platforms along with recently developed techniques to detect the most appropriate targets will enable potent antigen-specific humoral and cellular immune responses and will allow next-generation vaccines to be devised against a range of viral diseases, including SARS-CoV-2. For example, the COVID-19 mRNA (mRNA-1273) vaccine-loaded lipid nanoparticle (ionizable lipid:DSPC:cholesterol:PEG-lipid) can sufficiently induce neutralizing activity and CD8 T cell responses and protects against SARS-CoV-2 infection [256]. Translating materials science innovations into viable antiviral drugs and vaccines may create more efficient approaches and lead to clinically meaningful outcomes in the fight against COVID-19, with several bioactive nanomaterials currently in human clinical trials as a treatment against COVID-19 [207]. In addition, biomaterials can be engineered to not only deliver therapeutics such as anticoagulants via self-regulating mechanisms [257] but also designed with diagnostic imaging capabilities, potentially serving as translatable theranostic agents for thrombosis treatment [258,259].

Moreover, as COVID-19 infection can cause extensive multi-organ tissue damage, in addition to drug delivery and immunomodulation, biomaterials can be fabricated into biodegradable and porous scaffolds to promote cell migration and tissue regeneration [260].

### 5.3. Stem-Cell- and Tissue-Engineering-Based Therapies

Severe COVID-19 is characterized by damage or failure of multi-tissues and organs, including lung [17], cardiovascular system [261,262], nerve [263,264,265], kidney [266], liver [267], and intestine [268]. For example, SARS-CoV-2 infection facilitates the induction of endotheliitis/endothelial cell dysfunctions in several organs as a direct consequence of viral involvement and the host inflammatory response [261,269]. 

The application and/or in situ activation of stem cells can serve as an alternative therapeutic option to aid in tissue repair and reduce the mortality rate in critically ill COVID-19 patients [270,271,272,273]. While iPSCs can potentially be utilized in a personalized medicine approach, of particular interest are mesenchymal stem cells (MSCs), which can be derived from various sources (e.g., umbilical cord [273], bone marrow, dental pulp, adipose tissues [274], and menstrual blood [275]) and have been widely studied as a treatment in COVID-19 patients [276,277,278]. The immunomodulatory properties of these cells allow for inhibition of the cytokine storm while promoting tissue restoration [279]. They populate various adult tissues, avoiding invasive harvestation and moral issues, and are negative for ACE2 and TMPRSS2, so they are immune to invasion [279]. Upon activation of TLR receptors by pathogenic nucleic acids, MSCs release paracrine factors that work to rebalance lymphocyte populations [280]. If administered through IV infusion, the cells tend to accumulate in the lung, one of the hardest-hit organs, substantially improving its environment and preventing the onset of ARDS. Within a few days following MSC IV injection in patients whose symptoms were worsening, their fever, respiratory distress, and low oxygen levels improved or were relieved altogether with no observable side effects, and C-reactive protein levels decreased, confirming that the inflammation had diminished [279]. Long-term effects seen in this study included recovered liver and myocardial tissue. 

Stem cell nanoconjugate hybrid technology (for example, stem cells with a nanosponge or nanobubble delivery system) is currently being investigated as a nasal spray treatment against COVID-19 respiratory symptoms. Compared to stem cells alone, the hybrid nanoconjugates were shown to be more stable and biocompatible in the study; in general, the vehicles have higher loading capacity, longer half-life, and perform better against drug resistance. However, allogeneic MSCs risk exacerbating the harmful immune response, and autologous MSCs are not feasible as a blanket therapy for large numbers of COVID-19 patients due to the large expense and resources required [280]. Furthermore, long-term use of MSCs in COVID-19 patients in particular has yet to be investigated and declared safe. Therefore, because most benefits of MSC therapy stem from secreted regulatory nucleic acids and proteins rather than the cells themselves, a safe and practical option is to isolate their extracellular vesicles (EVs) and intravenously administer this cell-free solution [280]. EVs contain numerous bioactive molecules including RNAs, growth factors, proteins, and lipids that regulate distinct cellular processes (e.g., cell growth, proliferation, survival, and immune responses), thereby modulating various downstream pathways, including immunomodulation, suppression of apoptosis, prevention of fibrosis, and injured tissue remodeling. Stem-cell-derived exosomes can be used directly to alleviate tissue damage, as a drug-delivery system or potential vaccine for treating COVID-19 [278,281,282]. MSC-derived exosomes can elicit beneficial therapeutic effects in different conditions, including cardiovascular diseases, neurological diseases, kidney diseases, and wound healing [282,283]. Moreover, MSC-EVs-based therapy can inhibit influenza virus replication and virus-induced apoptosis in lung epithelial cells [284], suggesting that exosomes might be an effective therapeutic for COVID-19. EVs produced by cells engineered to express ACE2, and therefore present the viral receptor themselves, have been shown to prevent SARS-CoV-2 infection [252].

Furthermore, tissue-engineering-based technologies can be widely applied to the COVID-19 crisis through the use of in vitro models, drug delivery systems, vaccine platforms, and the repair of COVID-19-related damaged tissues [272]. At present, there are only a few preliminary clinical trials with small sample sizes that are testing stem cell products for COVID-19. For this reason, well-designed, multi-center randomized controlled trials are still needed to evaluate the efficiency and safety of stem-cell-based therapies for COVID-19. 

## 6. Challenges and Future Perspectives

With the advantages of rapid prototyping, cost-effective manufacturing, high reproducibility, and high flexibility in product design, 3D printing technology has the potential to play an important role in the field of biomedical engineering. In recent years, 3D printing has been applied to different biomedical-engineering-related areas, including but not limited to tissue and organ fabrication for regenerative medicine, 3D-printed drugs, customized prostheses, and cancer research [285]. In response to the recent COVID-19 pandemic, the application of 3D printing can aid with reducing the cost and relieving the shortage of PPE and medical equipment. With the rapid development of machine learning, recent research has shown that machine learning can also be implemented to help improve the efficiency and performance of the 3D printing process. Moreover, the shift from 3D to 4D printing, which utilizes smart materials and stimuli-responsive mechanisms to allow for dynamic and structural reconfiguration over time, holds great promise for the design of novel drug delivery systems, medical device fabrication, and regenerative medicine [285,286]. Yet, limitations such as slow print times, difficulty in printing at a large scale, lack of regulation, and no universal set of standards still need to be addressed before these 3D-printed devices are translated to clinical settings. Despite these limitations, 3D printing technology has the potential to benefit a wide range of bioengineering areas due to its promising features, in particular allowing for the development of highly personalized medicine. Furthermore, the increased accessibility of CAD will allow for 3D printing products to become readily available to the public and make a huge impact on the current manufacturing business in the foreseeable future.

Two widely accepted diagnostic methods for COVID-19 are RT-PCR and serology tests; however, both of these assays have limitations. RT-PCR is relatively more sensitive and accurate but requires a laboratory with proper equipment and well-trained personnel, which makes it expensive and difficult in medically underserved areas. In addition, sample transportation is required, and the multi-step protocol makes it a relatively slow process. Although serology tests can be performed in a relatively faster manner, sensitivity is relatively low and highly dependent on the time window after viral infection. Therefore, there is an urgent need for rapid, accurate, and low-cost diagnostic tests. PoC devices have the potential to address this need due to their aforementioned advantages (ASSURED) and can be easily expanded to diagnose other diseases beyond COVID-19. A potential way to reduce the cost is through 3D printing technology. For instance, a recent study developed a 3D-printed COVID-19 test chip platform that can rapidly detect SARS-CoV-2 antibodies and be regenerated within one minute for repeated use [287].

Currently, it is widely accepted that organ-on-a-chip provides a more accurate model than conventional cell culture for simulating complex cell–cell and cell–matrix interactions. To mimic the in vivo conditions more accurately and resolve the effect between different organs, techniques have been developed in recent years for the construction of organs-on-chips, which mainly consists of combining different chips together in order to study the integration of multiple organs within a system. These devices have attracted substantial interest, owing to their potential to be informative at multiple stages of the drug discovery and development processes [288]. Furthermore, these innovative devices could provide insights into normal human organ function and disease pathophysiology, as well as more accurately predict the safety and efficacy of investigational drugs in humans [288,289,290,291]. As COVID-19 induces blood vessel damage and coagulopathy, more commonly in patients with severe COVID-19, it is believed that endothelial cells are involved in these processes [292,293,294]. Thus, lung–blood vessels chips that incorporate both endothelial cells and immune cells (e.g., T cells and macrophages) should provide a powerful and efficient system to study virus/endothelial interactions, in addition to dose optimization during the screening of pharmaceutical agents. However, the combination of lung chips with blood vessel chips still remains a challenge and therefore a potential avenue for further development of organ-on-a-chip systems.

Several vaccines, including Pfizer–BioNTech and Moderna mRNA vaccines, and CoronaVac and Sinovac inactivated viral particles/vaccines, have been shown to be strongly effective and approved for clinical application [295,296]. The mRNA vaccines developed by Pfizer–BioNtech and Moderna use a lipid-based nanoparticle carrier system that prevents the rapid enzymatic degradation of mRNA and facilitates in vivo delivery. This lipid-based nanoparticle carrier system is further stabilized by a polyethylene glycol (PEG) 2000 lipid conjugate that provides a hydrophilic layer, prolonging half-life [297]. However, PEG or PEG derivatives as vaccine components may cause allergic reactions and adverse events, of which the underlying mechanisms need to be further elucidated [296]. In addition to the delivery system components, the design of these vaccine delivery methods could also be improved upon. Rather than having repeated bolus injections, it may be more efficient and cost-effective to utilize a platform that can deliver preprogrammed doses. For example, biodegradable materials can be implemented to allow for a burst release of the vaccine payload after 2-3 weeks in order to avoid a second visit and shot or even a sustained release of antigen to enhance immune responses. Moreover, the stability and storage of these vaccines are important parameters to consider. Improvements to the formulation to enhance mRNA vaccine stability, in particular at higher temperatures more suitable for vaccine distribution, are still necessary. Uncertainty in how long immune protection lasts and whether long-term immunity will need to be enhanced also requires further investigation. For example, a vaccine booster for more effective generation of T memory stem cells may promote long-term immunity, especially for the elderly and patients with immunosuppression. Progress in addressing these challenges will allow for the development of successful and effective vaccine strategies to combat infectious diseases, such as COVID-19.

Besides repurposed drugs, there are many new drug candidates in the pipeline [298]. One group screened 69 compounds by high-confidence protein–protein interactions between SARS-CoV-2 and human proteins using affinity purification–mass spectrometry and identified, in multiple viral assays, two sets of antiviral drugs: inhibitors of mRNA translation and regulators of sigma-1 and sigma-2 receptors [299]. Sheahan et al. demonstrated the broad-spectrum antiviral activity of NHC/EIDD-2801 against multiple CoVs, including SARS-CoV-2, MERS-CoV, SARS-CoV, and related zoonotic group 2b or 2c bat-CoVs, as well as remdesivir-resistant virus and multiple distinct zoonotic CoVs [300]. **GC373** and **GC376** are compounds capable of blocking SARS-CoV-2 virus replication [301]. Two compounds (**11a** and **11b**) exhibited excellent inhibitory activity and potent anti-SARS-CoV-2 infection activity by covalently binding to the cysteine 145 of main protease (M^pro^) [302]. While promising, extensive testing of these compounds is still necessary before they can be given to patients. Additionally, the development of safer and more efficient biomaterial-based platforms that take into consideration material design/fabrication, specific targeting, and controlled release will be beneficial for drug delivery, local immunomodulation, and tissue regeneration. Taken together, future advancements in micro/nano technologies and bioengineering approaches hold great promise in the fight against pandemic-causing infectious diseases, such as COVID-19.

## Figures and Tables

**Table 2:** Examples of serological tests used to detect SARS-CoV-2 antibodies.

Name of Serology Test	Type of Test	High Throughput	Sensitivity	Specificity	Type of Antibodies	PPV/NPV with 5% Prevalence Assumption of Infection in Sample Population	Limit of Detection or Smallest Volume Needed for Assay	Refs.
Abbott—Architect SARS-CoV-IgG	Chemiluminescent	Yes	100.0%	99.6%	IgG	93.4%/100%	Needs 100 μL of serum or plasma to be diluted	[73,74]
Babson Diagnostics aC19G1	Chemiluminescent	Yes	100.0%	100.0%	IgG	100%/100%	N/A	[75,76]
VITROS Anti-SARS-CoV-2 IgG test	Chemiluminescent	Yes	90.0%	100.0%	IgG	100%/99.5%	110 ng/well in assay with 20uL sample volume	[73,77]
EUROIMMUN	ELISA	Yes	90.0%	100.0%	IgG	100%/99.5%	10 μL of serum	[73]
WANTAI SARS-CoV-2 Ab ELISA	ELISA	No	96.7%	97.5%	Total antibody	67.1%/99.8%	100 μL needed of sample per test	[75,78]
Platelia SARS-CoV-2 Total Ab	ELISA	No	98.0%	99.3%	Total antibody	88.6%/99.9%	15 μL of specimen needed	[75,79]
Access BioCare Start COVID-19	Lateral Flow	No	98.4%	98.9%	IgG/IgM	82.5%/99.9%	N/A	[75,80]
RightSign COVID-19 IgG/IgM Rapid Test Cassette	Lateral Flow	No	100%	100%	IgG/IgM	100%/100%	~10 μL needed for assay	[75,81]
COVID-19 IgG/IgM Rapid Test Cassette	Lateral Flow	No	100.0%	97.5%	IgG/IgM	67.8%/100%	~10 μL for assay	[75,82]
Sienna-Clarity COVIBLOCK COVID-19 IgG/IgM Rapid Test Cassette	Lateral Flow	No	93.3%	98.8%	IgG/IgM	79.7%/99.6%	~10 μL	[75,83]

**Table 3:** Different types of vaccines.

Platform	Benefits	Drawbacks	Citations
mRNA	Reduced genotoxicity compared to DNA vaccines Amenable for quality control with reduced probability of biological contamination compared to inactivated virus or live vector-based vaccines Relatively quick design and manufacture process to meet pandemic needs	Unstable without proper formulation Easily degraded leading to reduced dose efficacy Potential activation of innate response resulting in poor patient outcomes Difficult transportation method requiring cold-chain conditions for storage Specific target thus low coverage for viral mutations	[208,209,210,211,212,213,214]
DNA	Quick production, taking approximately 2 to 4 weeks Readily and quickly denatures under many conditions	Potential activation of oncogenes Potential autoimmune response activation and chronic inflammation Limited immunogenicity in animal models Specific target thus low coverage for viral mutations	[215,216,217,218]
Recombinant Protein	Historically effective vaccine mechanism Reduced risk of genotoxicity and/or chronic inflammation due to dose-dependent effects	More extensive production and purification steps required Multiple dosing regimens with adjuvants to achieve effective responses Broad targets, however, often low responses	[219,220,221]
Virus-like Particles	Maintain the conformation of viral proteins improving efficacy Reduced risk of genotoxicity and/or chronic inflammation due to dose-dependent effects	More intensive process of manufacturing (including more detailed steps than recombinant proteins) Multiple dosing regimens expected, similar to recombinant proteins	[222,223,224]
Peptide	Smaller size and more stable structure compared to larger proteins	Often ineffective levels of immune response due to short length of amino acid sequences Specific target thus low coverage for viral mutations	[225,226,227]

## Data Availability

Not applicable.
